# Analysing the Applicability of ChatGPT, Bard, and Bing to Generate Reasoning-Based Multiple-Choice Questions in Medical Physiology

**DOI:** 10.7759/cureus.40977

**Published:** 2023-06-26

**Authors:** Mayank Agarwal, Priyanka Sharma, Ayan Goswami

**Affiliations:** 1 Physiology, All India Institute of Medical Sciences, Raebareli, IND; 2 Physiology, School of Medical Sciences and Research, Sharda University, Greater Noida, IND; 3 Physiology, Santiniketan Medical College, Bolpur, IND

**Keywords:** physiology, medical education, examination questions, educational technology, artificial intelligence

## Abstract

Background

Artificial intelligence (AI) is evolving in the medical education system. ChatGPT, Google Bard, and Microsoft Bing are AI-based models that can solve problems in medical education. However, the applicability of AI to create reasoning-based multiple-choice questions (MCQs) in the field of medical physiology is yet to be explored.

Objective

We aimed to assess and compare the applicability of ChatGPT, Bard, and Bing in generating reasoning-based MCQs for MBBS (Bachelor of Medicine, Bachelor of Surgery) undergraduate students on the subject of physiology.

Methods

The National Medical Commission of India has developed an 11-module physiology curriculum with various competencies. Two physiologists independently chose a competency from each module. The third physiologist prompted all three AIs to generate five MCQs for each chosen competency. The two physiologists who provided the competencies rated the MCQs generated by the AIs on a scale of 0-3 for validity, difficulty, and reasoning ability required to answer them. We analyzed the average of the two scores using the Kruskal-Wallis test to compare the distribution across the total and module-wise responses, followed by a post-hoc test for pairwise comparisons. We used Cohen's Kappa (Κ) to assess the agreement in scores between the two raters. We expressed the data as a median with an interquartile range. We determined their statistical significance by a p-value <0.05.

Results

ChatGPT and Bard generated 110 MCQs for the chosen competencies. However, Bing provided only 100 MCQs as it failed to generate them for two competencies. The validity of the MCQs was rated as 3 (3-3) for ChatGPT, 3 (1.5-3) for Bard, and 3 (1.5-3) for Bing, showing a significant difference (p<0.001) among the models. The difficulty of the MCQs was rated as 1 (0-1) for ChatGPT, 1 (1-2) for Bard, and 1 (1-2) for Bing, with a significant difference (p=0.006). The required reasoning ability to answer the MCQs was rated as 1 (1-2) for ChatGPT, 1 (1-2) for Bard, and 1 (1-2) for Bing, with no significant difference (p=0.235). K was ≥ 0.8 for all three parameters across all three AI models.

Conclusion

AI still needs to evolve to generate reasoning-based MCQs in medical physiology. ChatGPT, Bard, and Bing showed certain limitations. Bing generated significantly least valid MCQs, while ChatGPT generated significantly least difficult MCQs.

## Introduction

A precise definition of artificial intelligence (AI) is not available [1]. Still, AI refers to designing and developing computer systems that emulate human cognitive functioning to solve problems or conduct complex tasks. AI is currently the subject of extensive research across various scientific fields and is rapidly advancing in healthcare and biomedical research [2,3]. AI’s partial cognitive abilities include but are not limited to problem-solving, reasoning, and decision-making [4,5]. A noteworthy AI-based conversational tool, ChatGPT, is freely available for research. The other AIs are Microsoft Bing and Google Bard (currently in the experimental phase).

Medical physiology is of great significance in medical education as it provides a comprehensive understanding of the complex and intricate workings of the human body at macroscopic and microscopic levels [6]. A strong comprehension of physiology is essential for medical students to appreciate the pathophysiology of a disease. Students can identify diseased states and conclude therapeutic approaches by elucidating the underlying physiological mechanisms that regulate bodily functions.

Creating reasoning-based multiple-choice questions (MCQs) requires high cognitive skills, such as identifying key concepts and facts, applying the information for developing logical options, drawing conclusions from those options, and evaluating the final MCQ [7]. The creator of the MCQs must have a good understanding of the subject matter to support their views in constructing a compelling question that can accurately assess the examinee’s knowledge [7].

Previous studies have shown that ChatGPT can solve higher-order questions, including the United States Medical Licensing Examination (USMLE), which indicates the presence of a few cognitive capabilities in ChatGPT, such as logic and reasoning [8-12]. Thus, we hypothesised that AI could generate reasoning-based MCQs in medical physiology.

The current study aimed to assess and compare the applicability of ChatGPT, Google Bard, and Microsoft Bing in generating reasoning-based MCQs. The findings of this study would provide an understanding of the strengths and weaknesses of AI as an automated MCQ generation system in the context of medical physiology. Additionally, the results would serve as a foundation for future research endeavors in this field, contributing to advancements in the automated MCQ generation for medical education.

## Materials and methods

Study setting and ethical consideration

This cross-sectional study was conducted during the first and second week of June 2023. The study data were collected from open-source and recent versions of ChatGPT (version May 24, 2023), Google Bard (version June 1, 2023), and Microsoft Bing AI. Since the study does not involve human or animal research subjects, there was no requirement for an ethical review by the institutional review board according to standard guidelines.

Data collection

As part of its Competency-Based Medical Education (CBME) framework, the Indian National Medical Commission (NMC) has developed an 11-module curriculum for physiology. Furthermore, the NMC has subdivided each of these 11 modules into various competencies. The primary objective of this curriculum is to assess the student’s comprehension of the subject matter, focussing on understanding rather than just the accumulation of factual knowledge [13].

The study involved three experienced physiologists from different medical colleges in India, each with over four years of teaching experience after completing their postgraduation. Two physiologists independently chose one competency of the ‘know-how’ domain from each module. The third physiologist then compiled the competencies provided by the other two and used AIs to generate five complex reasoning-based MCQs for MBBS (Bachelor of Medicine, Bachelor of Surgery) undergraduates. All three physiologists examined the prompts for face and content validity and approved them. Table 1 lists the prompts for the MCQs generation for each chosen competency from the 11 modules of the NMC CBME curriculum.

**Table 1:** Modules of medical physiology according to the NMC CBME curriculum and prompts given to AIs for the generation of MCQs AI, Artificial Intelligence; CBME, Competency-Based Medical Curriculum; MBBS, Bachelor of Medicine and Bachelor of Surgery; MCQs, Multiple Choice Questions; NMC, National Medical Commission

Module Name	Prompts used for MCQs generation
PY 1: General Physiology	Generate 5 difficult reasoning-based MCQs for MBBS undergraduates on the mechanism of transport across the cell membrane
	Generate 5 difficult reasoning-based MCQs for MBBS undergraduates on the ionic basis of resting membrane potential and action potential
PY 2: Blood Physiology	Generate 5 difficult reasoning-based MCQs for MBBS undergraduates on the physiology of hemostasis
	Generate 5 difficult reasoning-based MCQs for MBBS undergraduates on the physiology of blood grouping
PY 3: Nerve and Muscle Physiology	Generate 5 difficult reasoning-based MCQs for MBBS undergraduates on the physiology of neuromuscular junction
	Generate 5 difficult reasoning-based MCQs for MBBS undergraduates on the ionic and molecular basis of skeletal muscle contraction
PY 4: Gastrointestinal Physiology	Generate 5 difficult reasoning-based MCQs for MBBS undergraduates on the physiology of gastrointestinal movements
	Generate 5 difficult reasoning-based MCQs for MBBS undergraduates on the physiology of gastrointestinal secretions
PY 5: Cardiovascular Physiology	Generate 5 difficult reasoning-based MCQs for MBBS undergraduates on the regulation of blood pressure
	Generate 5 difficult reasoning-based MCQs for MBBS undergraduates on an ionic basis of cardiac impulse generation
PY 6: Respiratory Physiology	Generate 5 difficult reasoning-based MCQs for MBBS undergraduates on the physiology of high altitude and deep sea diving
	Generate 5 difficult reasoning-based MCQs for MBBS undergraduates on the physiology of transport of respiratory gases
PY 7: Renal Physiology	Generate 5 difficult reasoning-based MCQs for MBBS undergraduates on the physiology of glomerular filtration
	Generate 5 difficult reasoning-based MCQs for MBBS undergraduates on the physiology of acid-base balance by kidneys
PY 8: Endocrine Physiology	Generate 5 difficult reasoning-based MCQs for MBBS undergraduates on the physiology of growth hormone
	Generate 5 difficult reasoning-based MCQs for MBBS undergraduates on the physiology of thyroid hormone
PY 9: Reproductive Physiology	Generate 5 difficult reasoning-based MCQs for MBBS undergraduates on the physiology of parturition and lactation
	Generate 5 difficult reasoning-based MCQs for MBBS undergraduates on the physiology of contraception
PY 10: Neurophysiology	Generate 5 difficult reasoning-based MCQs for MBBS undergraduates on the physiology of the autonomic nervous system
	Generate 5 difficult reasoning-based MCQs for MBBS undergraduates on the physiology of vision
PY 11: Integrated Physiology	Generate 5 difficult reasoning-based MCQs for MBBS undergraduates on cardiorespiratory and metabolic adjustments during exercise
	Generate 5 difficult reasoning-based MCQs for MBBS undergraduates on the physiology of temperature regulation

Three AI models, namely, ChatGPT, Bard, and Bing, simultaneously provided responses on June 2, 2023. The first response from each AI was taken as the final, and no regeneration option was used. The MCQs generated by the AI models were copied and pasted into an Excel spreadsheet for subsequent analyses.

The two physiologists, who provided the competencies, were tasked with rating the three sets of 110 MCQs each on three parameters - validity, difficulty, and reasoning ability. As shown in Table 2, the rating scale ranged from 0 to 3, where 0 indicated the lowest and 3 indicated the highest validity, difficulty, or reasoning ability.

**Table 2:** Rating used for MCQ analysis MCQ, Multiple Choice Question

Parameter	Rating
MCQ is valid (acceptable) for the competency of the medical physiology subject. Moreover, the stem and options of the MCQs are clear without any ambiguity.	0: Not valid
	1: Somewhat valid
	2: Valid
	3: Highly valid
Difficulty level of MCQ	0: Not difficult or very easy
	1: Somewhat difficult or easy
	2: Difficult
	3: Very difficult
Reasoning/understanding of the subject required to solve MCQ	0: Not required (factual question)
	1: Somewhat required
	2: Required
	3: High level of understating required

**Figure 1:**
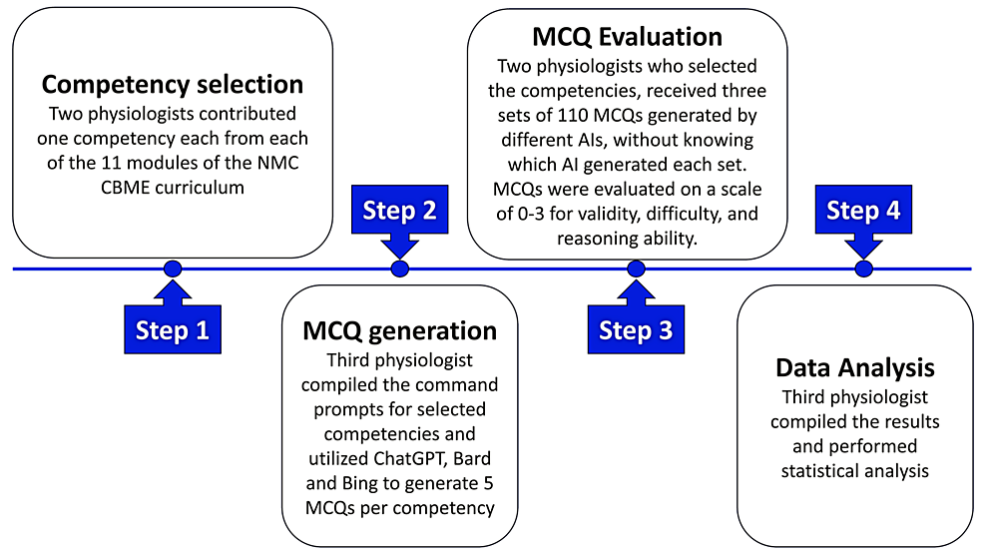
Brief outline of the method followed in the study AI, Artificial Intelligence; CBME, Competency-Based Medical Curriculum; MCQs, Multiple Choice Questions; NMC, National Medical Commission

The physiologists were blinded, which meant they were unaware of which AI model generated each set of MCQs. After a week of analysis, the physiologists returned the results to the third physiologist, who compiled the data and applied statistical methods for further analysis. The ratings provided by the two physiologists were averaged to obtain the final results for validity, difficulty, and reasoning ability. Additionally, Turnitin software was used to check the originality of the content for all sets of MCQs. Figure 1 briefly outlines the method used in the study.

Statistical analysis

We initially entered the data using Microsoft Excel 365 and then performed the statistical analysis in IBM SPSS Statistics for Windows Version 27.0. Since the data were ordinal, we employed non-parametric tests. We presented the data as medians with an interquartile range (Q1-Q3). To compare the distribution across the total and module-wise responses, we utilized the independent sample Kruskal-Wallis test, followed by a post-hoc test, for pairwise comparisons. We assessed the agreement in scores between the two raters using Cohen’s Kappa (Κ). We determined the statistical significance by a p-value <0.05.

## Results

ChatGPT and Bard provided 110 MCQs for 22 chosen competencies from the 11 modules of the NMC CBME curriculum for physiology. However, Bing provided only 100 MCQs as it failed to generate them for two competencies - physiology of blood grouping and ionic and molecular basis of skeletal muscle contraction. For these two competencies, Bing was given a ‘0’ rating for all three parameters - validity, difficulty, and reasoning ability. Overall, Bing generated the least valid MCQs, and ChatGPT generated the least difficult MCQs, as shown in Table 3.

**Table 3:** Overall scores of the MCQs generated by AIs AI, Artificial Intelligence; MCQs, Multiple Choice Questions Only the significant post-hoc p-values are shown for p<0.05 in the Kruskal-Wallis test

N= 110	ChatGPT		Bard		Bing		p-value
	Median (Q1-Q3)	Cohen’s K	Median (Q1-Q3)	Cohen’s K	Median (Q1-Q3)	Cohen’s K	
Validity	3 (3-3)	0.847	3 (1.5-3)	0.848	3 (1.5-3)	0.833	<0.001 (ChatGPT vs Bing <0.001; Bard vs Bing <0.001)
Difficulty	1 (0-1)	0.803	1 (1-2)	0.818	1 (1-2)	0.802	0.006 (ChatGPT vs Bing 0.010; ChatGPT vs Bard 0.003)
Reasoning ability	1 (1-2)	0.802	1 (1-2)	0.811	1 (1-2)	0.798	0.235

The Turnitin test similarity indices for ChatGPT, Bard, and Bing were 39, 49, and 52%, respectively. A significantly (p<0.001) strong inter-rater reliability (Cohen’s K ≥ 0.8) was obtained for all three parameters for all three AIs.

Module-wise scores (Table 4) showed that Bing generated the least valid and least difficult MCQs for blood physiology, the least valid MCQs for nerve-muscle physiology, and the most difficult MCQs for reproductive physiology. ChatGPT generated the least difficult MCQs for cardiovascular physiology, the most valid and reasoning-based MCQs for endocrine physiology, and the most valid MCQs for integrated physiology. Bard generated the least valid MCQs for respiratory physiology. Post-hoc p-values are shown only when the Kruskal-Wallis test p-value is statistically significant.

**Table 4:** Module-wise scores of the MCQs generated by AIs AI, Artificial Intelligence; MCQs, Multiple Choice Questions Only the significant post-hoc p-values are shown for p<0.05 in the Kruskal-Wallis test

Module (N=10 for each module)	Parameter	ChatGPT	Bard	Bing	p-value
		Median (Q1-Q3)	Median (Q1-Q3)	Median (Q1-Q3)	
General Physiology	Validity	3 (3-3)	3 (3-3)	3 (3-3)	1.000
	Difficulty	1 (0-1)	1 (0.75-2)	1 (0-1)	0.128
	Reasoning ability	1 (0.875-1.625)	2 (1-2)	0.5 (0-2)	0.103
Blood Physiology	Validity	3 (3-3)	3 (2-3)	0 (0-2.625)	<0.001 (ChatGPT vs Bing 0.001; Bard vs Bing 0.021)
	Difficulty	0.5 (0-1)	2 (0.75-2.625)	0 (0-1.25)	0.028 (ChatGPT vs Bard 0.023; Bard vs Bing 0.019)
	Reasoning ability	1 (1-2)	2 (1-2)	0 (0-2)	0.061
Nerve and Muscle Physiology	Validity	3 (3-3)	3 (2.75-3)	0.5 (0-3)	0.005 (ChatGPT vs Bing 0.002; Bard vs Bing 0.018)
	Difficulty	1 (0.375-1)	1 (0.375-1.25)	0.5 (0-1.5)	0.760
	Reasoning ability	1.25 (0.875-2)	1 (1-1.25)	0.5 (0-1.625)	0.254
Gastrointestinal Physiology	Validity	3 (3-3)	3 (2.5-3)	3 (3-3)	0.126
	Difficulty	1.25 (1-2)	1 (1-2)	1 (0.75-1.125)	0.198
	Reasoning ability	1 (1-1.25)	1.25 (1-2)	1 (0-1.125)	0.171
Cardiovascular Physiology	Validity	3 (2.875-3)	2.25 (1.875-3)	3 (2.625-3)	0.105
	Difficulty	0 (0-0.625)	1.25 (0.75-2)	1.5 (1-2)	0.005 (ChatGPT vs Bard 0.020; ChatGPT vs Bing 0.002)
	Reasoning ability	1.25 (1-2)	2 (1-2)	1 (1-2)	0.201
Respiratory Physiology	Validity	3 (3-3)	2 (1-3)	3 (2-3)	0.010 (ChatGPT vs Bard 0.003)
	Difficulty	1 (0.375-1.125)	1.25 (1-2)	1 (1-1.125)	0.085
	Reasoning ability	1 (1-2)	1.75 (1-2)	1.75 (1-2)	0.522
Renal Physiology	Validity	2.75 (1.75-3)	3 (2.375-3)	3 (2-3)	0.653
	Difficulty	1.25 (0.875-2)	1.5 (1-2)	1 (1-3)	0.840
	Reasoning ability	2 (1.875-2)	2 (1.75-2)	1.75 (1-3)	0.943
Endocrine Physiology	Validity	3 (3-3)	2.75 (1-3)	1.75 (1-3)	0.009 (ChatGPT vs Bard 0.025; ChatGPT vs Bing 0.003)
	Difficulty	1 (0.75-1.125)	1 (1-1.125)	1.25 (1-2.625)	0.092
	Reasoning ability	1.25 (1-2)	1 (0-1)	1 (1-2)	0.023 (ChatGPT vs Bard 0.012; Bard vs Bing 0.026)
Reproductive Physiology	Validity	3 (1-3)	1.25 (1-2)	2.25 (1.375-3)	0.155
	Difficulty	1 (0.375-1)	1 (1-1)	1.5 (1-2)	0.011 (ChatGPT vs Bing 0.004; Bard vs Bing 0.033)
	Reasoning ability	1 (1-2)	1 (0.874-1.125)	1 (1-1.5)	0.479
Neurophysiology	Validity	3 (3-3)	2.75 (0.75-3)	3 (1.75-3)	0.124
	Difficulty	0 (0-1)	0 (0-1.125)	1 (0.5-2)	0.083
	Reasoning ability	1 (1-1.125)	0.5 (0-1.25)	1 (0.875-1.625)	0.236
Integrated Physiology	Validity	3 (3-3)	1.75 (1-3)	2 (1-2.625)	0.002 (ChatGPT vs Bing 0.001; ChatGPT vs Bard 0.003)
	Difficulty	1 (1-2)	1 (0.375-2)	1 (0.875-2)	0.756
	Reasoning ability	2 (1-2)	2 (0.75-2)	2 (1.375-2)	0.853

Although the timings were not recorded, ChatGPT seemed to be the slowest among the three AIs to generate MCQs. Bard generated 47 MCQs in which stem had the suffix ‘Which of the following is the most important,’ and 54 MCQs had ‘all of the above’ as an option. Bard was the only AI that provided answers to the MCQs with an explanation. In all the generated MCQs by Bing, the stem of the questions consistently had the suffix ‘Which of the following is not,’ indicating a negative verb construction.

## Discussion

The current study assessed the applicability of ChatGPT, Bard, and Bing in generating valid, difficult, and reasoning-based MCQs in medical physiology. The results show that ChatGPT generated the most valid MCQs but the least difficult ones. None of the three AIs could generate a considerable number of MCQs that required a high level of subject understanding (reasoning ability).

During the analysis of MCQs, we identified a few shortcomings. The MCQs created by Bing and Bard did not fulfill the criteria of an ideal MCQ. A good MCQ should not contain negative words in the stem and ‘all of the above’ as an option in the choices [14,15]. Moreover, the stem of MCQ asking for ‘most important’ often emphasizes on factual knowledge rather than comprehension. Moreover, it was observed that ChatGPT generated MCQs with the lowest text similarity index, while Bing had the highest text similarity index.

Among the three AIs, a significant difference in a few scores across different competency modules was observed, which could be attributed to the limited training of the AI systems. The choice of words for prompts could be another reason for the different levels of AI performance. There is scope for further improvement in AI models to make them more suitable for educational use.

Recent studies conducted by medical professionals in India have shown that the ChatGPT is a reliable tool for solving problems that require higher-level thinking, interpretation, analysis, evaluation, or formulation of opinions and predictions based on evidence in pathology, biochemistry, and microbiology [8-10]. In addition, ChatGPT was proficient in answering straightforward queries seeking factual information in microbiology [8]. Another study evaluated the capabilities of ChatGPT in answering NMC CBME-based medical physiology question paper of 100 marks that included two essays (15 marks each), 10 short notes (5 marks each), and 20 MCQs. Overall, ChatGPT performed exceptionally well in the Physiology University Examination, achieving a distinction by obtaining more than 75% of the marks [16].

Other previous studies investigating the potential of ChatGPT for medical education applications have reported that ChatGPT not only possesses a remarkable ability to provide accurate responses to medical inquiries, but also its performance was comparable to that of a third-year medical student in the United States [11]. ChatGPT not only exhibited the capacity to pass the USMLE independently without any human assistance but also showcased comprehensible reasoning and provided valid clinical insights in its responses [11,12]. ChatGPT performed well at the German state licensing exam level in Progress Test Medicine by correctly answering two-thirds of the MCQs, outperforming most medical students in their first to third year of study [17]. According to the assessment of 33 physicians spanning 17 specialties, ChatGPT mostly generated accurate and complete information in response to 284 varied medical questions [18].

However, in the current study, when tasked with creating reasoning-based MCQs that require a deep understanding of physiology, the capabilities of AI fall short of human intelligence. Comparable to our study results, a recent study indicated that ChatGPT’s intelligence was lower than the Korean medical students for the parasitology examination [19].

The integration of technology into medical education presents exciting opportunities for innovation. AI can offer answers and explanations related to medical topics in an easily accessible and comprehensible manner. Refraining from considering the use of AI in medical education is no longer a practical choice. The focus has shifted from whether students and faculty will use AI to when and how they will use it [20].

We suggest that medical colleges should take advantage of AI and curate carefully designed, developed, and validated AI systems to extract accurate and trustworthy information. The utilization of automatically generated reasoning-based MCQs by AI during live lectures has the potential to revolutionize medical education. This approach would improve the interaction between teachers and students as both would encounter the questions simultaneously, which could transform the lecture into a more dynamic and engaging learning experience.

However, despite AI's current advantages and future potential, ensuring the accuracy and reliability of the information provided by these systems is still a primary challenge. At present, Bard and ChatGPT outperform Bing in creating valid MCQs, but the same cannot be said for the future as AIs are evolving rapidly. A recent study has indicated that ChatGPT was superior to Bard in answering higher-order questions for neurosurgery oral board preparation [21]. Compared to Bard and Bing, ChatGPT produced more accurate and consistent responses to non-expert queries about lung cancer prevention, screening, and terminology [22].

Limitations

This study had several limitations. We solely focused on evaluating the applicability of AI systems to generate MCQs related to medical physiology. Therefore, the findings and conclusions drawn from this study might not be applicable or generalizable to other subjects or domains. Another limitation of the study is the reliance on a single user for conversational interactions with the ChatGPT. Conversations with different users may yield varying responses, particularly at different time points. Additionally, we acknowledge that paraphrasing questions can introduce variations in the responses provided by ChatGPT, which could impact the overall evaluation of the system’s performance. The scoring of the AI-generated responses in our study was subjective, as it relied on human evaluators. Despite our efforts to mitigate bias by averaging the scores of the two evaluators, the presence of evaluation bias cannot be completely ruled out. Finally, we could not involve MBBS students in the item analysis of the MCQs [23].

## Conclusions

Our study provides valuable insights into the applicability of AI systems to generate reasoning-based MCQs in the medical physiology domain. The results of this study indicate that AI’s abilities to create reasoning-based MCQs are not yet fully developed. All three AI models, namely, ChatGPT, Bard, and Bing, showed certain limitations, and there is still room for improvement. ChatGPT and Bard were marginally better than Bing in MCQ creation. However, the current study results should be interpreted with caution because of the limitations of the research design. Future studies should address the present study’s limitations and expand the investigation’s scope to yield more robust and generalizable findings.

## Figures and Tables

**Figure 2:**
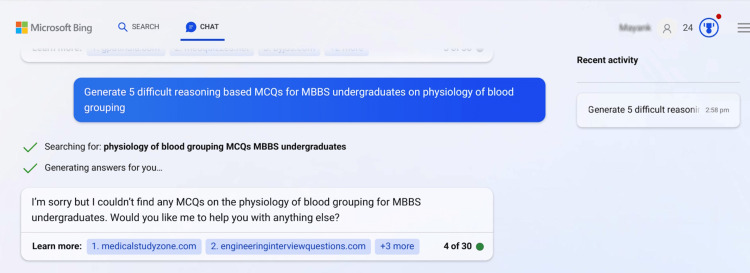
Partial screenshot of a conversation with Bing. Bing failed to generate five MCQs on the physiology of blood grouping MCQS, Multiple Choice Questions To ensure anonymity and enable blind peer review, the registered user's name has been blurred.

**Figure 3:**
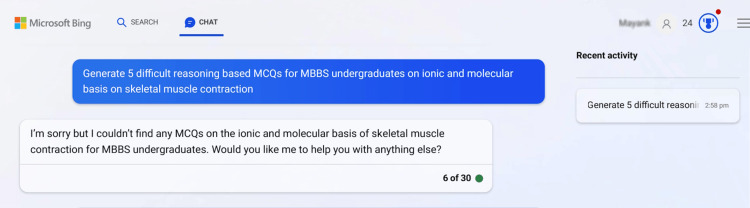
Partial screenshot of a conversation with Microsoft Bing. Bing failed to generate five MCQs on the ionic and molecular basis of skeletal muscle contraction MCQs, Multiple Choice Questions To ensure anonymity and enable blind peer review, the registered user's name has been blurred

## Appendices

**Table 5:** MCQs generated by Microsoft Bing, Google Bard, and ChatGPT MCQs, Multiple Choice Questions Disclaimer: The MCQs exhibit a high text similarity index as determined by Turnitin software. These questions have been shared with the intention of upholding the transparency of the conducted research

Bing	Bard	ChatGPT
General Physiology		
Which of the following is not a type of passive transport? a) Simple diffusion b) Facilitated diffusion c) Osmosis d) Active transport	Which of the following transport mechanisms requires the use of energy? (A) Facilitated diffusion (B) Active transport (C) Simple diffusion (D) Osmosis	Which of the following mechanisms is responsible for the active transport of ions across the cell membrane? a) Simple diffusion b) Facilitated diffusion c) Osmosis d) Sodium-potassium pump
Which of the following is not a type of active transport? a) Primary active transport b) Secondary active transport c) Facilitated diffusion d) Endocytosis	Which of the following molecules would be most likely to diffuse across a cell membrane? (A) Glucose (B) Sodium (C) Potassium (D) Chloride	Which of the following processes requires the expenditure of energy to transport molecules across the cell membrane? a) Passive transport b) Osmosis c) Simple diffusion d) Active transport
Which of the following is not a type of endocytosis? a) Phagocytosis b) Pinocytosis c) Receptor-mediated endocytosis d) Exocytosis	Which of the following transport mechanisms is used to move glucose into cells from the bloodstream? (A) Facilitated diffusion (B) Active transport (C) Simple diffusion (D) Osmosis	Which of the following best describes the process of endocytosis? a) Movement of water molecules across a selectively permeable membrane b) Diffusion of molecules from an area of higher concentration to an area of lower concentration c) Uptake of large molecules or particles into the cell by the formation of vesicles d) Transport of molecules across the membrane with the help of carrier proteins
Which of the following is not a type of exocytosis? a) Constitutive exocytosis b) Regulated exocytosis c) Transcytosis d) Endocytosis	Which of the following transport mechanisms is used to move sodium out of cells and potassium into cells? (A) Facilitated diffusion (B) Active transport (C) Simple diffusion (D) Osmosis	Which of the following transport mechanisms is responsible for the movement of glucose molecules into the cell against its concentration gradient? a) Simple diffusion b) Facilitated diffusion c) Active transport d) Exocytosis
Which of the following is not a type of facilitated diffusion? a) Channel-mediated facilitated diffusion b) Carrier-mediated facilitated diffusion c) Osmosis d) None of the above	Which of the following is a difference between facilitated diffusion and active transport? (A) Facilitated diffusion requires energy, while active transport does not. (B) Facilitated diffusion is a passive process, while active transport is an active process. (C) Facilitated diffusion can move molecules against their concentration gradient, while active transport cannot. (D) Facilitated diffusion requires a carrier protein, while active transport does not.	Which of the following statements about the sodium-potassium pump is correct? a) It transports sodium ions out of the cell and potassium ions into the cell. b) It functions through simple diffusion across the cell membrane. c) It is an example of passive transport. d) It does not require the expenditure of energy.
Which of the following is not a type of ion channel? a) Voltage-gated ion channels b) Ligand-gated ion channels c) Mechanically gated ion channels d) None of the above	Which of the following ions is the major determinant of the resting membrane potential? (A) Sodium (B) Potassium (C) Chloride (D) Calcium	Which ion primarily contributes to the resting membrane potential of a neuron? a) Sodium (Na+) b) Potassium (K+) c) Chloride (Cl-) d) Calcium (Ca2+)
Which of the following is not a type of ion that contributes to the resting membrane potential? a) Sodium ions b) Potassium ions c) Chloride ions d) Calcium ions	Which of the following channels is responsible for the rapid influx of sodium ions during an action potential? (A) Na+,K+-ATPase (B) Voltage-gated sodium channels (C) Leaky potassium channels (D) Inwardly rectifying potassium channels	The resting membrane potential of a neuron is typically maintained at approximately: a) -70 mV b) +30 mV c) 0 mV d) -40 mV
Which of the following is not a type of ion that contributes to the action potential? a) Sodium ions b) Potassium ions c) Chloride ions d) Calcium ions	Which of the following channels is responsible for the delayed outward potassium current that terminates an action potential? (A) Na+,K+-ATPase (B) Voltage-gated sodium channels (C) Leaky potassium channels (D) Inwardly rectifying potassium channels	Which ion channels are responsible for the depolarization phase of an action potential? a) Voltage-gated sodium channels b) Voltage-gated potassium channels c) Ligand-gated sodium channels d) Ligand-gated potassium channels
Which of the following is not a phase of the action potential? a) Depolarization phase b) Repolarization phase c) Hyperpolarization phase d) Resting phase	Which of the following factors would increase the likelihood of an action potential being generated? (A) A decrease in the extracellular sodium concentration (B) An increase in the extracellular potassium concentration (C) A decrease in the resting membrane potential (D) A decrease in the number of voltage-gated sodium channels	During the repolarization phase of an action potential, which ion channels open? a) Voltage-gated sodium channels b) Voltage-gated potassium channels c) Ligand-gated sodium channels d) Ligand-gated potassium channels
Which of the following is not a type of voltage-gated ion channel? a) Sodium channels b) Potassium channels c) Calcium channels d) Chloride channels	Which of the following conditions would prevent an action potential from being generated? (A) A complete block of voltage-gated sodium channels (B) A complete block of leaky potassium channels (C) A complete block of inwardly rectifying potassium channels (D) A complete block of Na+,K+-ATPase	Which of the following best describes the all-or-nothing principle of an action potential? a) The magnitude of an action potential varies depending on the strength of the stimulus. b) An action potential can be generated by a sub-threshold stimulus. c) Once the threshold is reached, an action potential is generated with a fixed magnitude. d) An action potential can be generated in both directions along the axon.
Blood Physiology		
Which of the following is not a component of hemostasis? a) Vascular spasm b) Platelet plug formation c) Coagulation d) Fibrinolysis	Which of the following is the first step in hemostasis? (A) Platelet aggregation (B) Coagulation cascade (C) Fibrinolysis (D) Vascular spasm	Which of the following is the primary role of platelets in hemostasis? a) Formation of a fibrin clot b) Activation of the coagulation cascade c) Vasoconstriction of injured blood vessels d) Recruitment of white blood cells to the site of injury
Which of the following is not a type of platelet receptor? a) GPIb-IX-V b) GPVI c) GPCRs d) Integrins	Which of the following is the most important platelet agonist? (A) Thrombin (B) ADP (C) Fibrinogen (D) Calcium	Which of the following factors is responsible for initiating the intrinsic pathway of coagulation in hemostasis? a) Factor VII b) Factor VIII c) Factor IX d) Factor XII
Which of the following is not a coagulation factor? a) Factor I (fibrinogen) b) Factor II (prothrombin) c) Factor III (tissue factor) d) Factor IV (calcium)	Which of the following is the most important factor in the coagulation cascade? (A) Factor XII (B) Factor VII (C) Factor VIII (D) Factor IX	Which of the following is NOT a function of antithrombin III in hemostasis? a) Inhibition of thrombin activity b) Inhibition of factor Xa activity c) Activation of plasminogen d) Inhibition of factors IXa and XIa
Which of the following is not a type of anticoagulant? a) Heparin b) Warfarin c) Aspirin d) Clopidogrel	Which of the following is the most important inhibitor of the coagulation cascade? (A) Antithrombin III (B) Protein C (C) Protein S (D) Tissue factor pathway inhibitor	Which of the following is the primary physiological mechanism for fibrinolysis in hemostasis? a) Activation of tissue plasminogen activator (tPA) b) Activation of factor XII c) Activation of factor VIII d) Activation of factor XIII
Which of the following is not a type of hemophilia? a) Hemophilia A b) Hemophilia B c) Hemophilia C d) Hemophilia D	Which of the following is the most important fibrinolytic enzyme? (A) Plasminogen activator (B) Plasmin (C) Alpha-2-antiplasmin (D) Thrombin	Which of the following conditions is characterized by a deficiency of von Willebrand factor (vWF) and results in impaired platelet adhesion in hemostasis? a) Hemophilia A b) Hemophilia B c) von Willebrand disease d) Thrombocytopenia
n/a	A patient with type A blood is transfused with type B blood. What is the most likely outcome of this transfusion? (A) The patient will develop anaphylaxis. (B) The patient will develop a hemolytic reaction. (C) The patient will develop a febrile reaction. (D) The patient will not experience any adverse effects.	Which of the following blood groups is considered to be the universal donor in terms of red blood cell transfusion? a) A positive b) B negative c) O positive d) AB negative
n/a	A patient with type AB blood is pregnant with a fetus that has type O blood. What is the most likely outcome of this pregnancy? (A) The fetus will be at risk for hemolytic anemia. (B) The fetus will be at risk for anaphylaxis. (C) The fetus will be at risk for a febrile reaction. (D) The fetus will not experience any adverse effects.	The presence of which antigen on the surface of red blood cells determines the Rh-positive blood group? a) A antigen b) B antigen c) D antigen d) O antigen
n/a	A patient with type O blood is a universal donor. What does this mean? (A) The patient's blood can be transfused into any patient, regardless of their blood type. (B) The patient's blood can only be transfused into patients with type O blood. (C) The patient's blood can only be transfused into patients with type AB blood. (D) The patient's blood can only be transfused into patients with type A or B blood.	A person with blood group AB positive can receive blood from which of the following donors? a) A negative b) B positive c) O negative d) AB negative
n/a	A patient with type AB blood is a universal recipient. What does this mean? (A) The patient can receive blood from any donor, regardless of their blood type. (B) The patient can only receive blood from donors with type O blood. (C) The patient can only receive blood from donors with type AB blood. (D) The patient can only receive blood from donors with type A or B blood.	In the ABO blood group system, which antibodies are naturally present in the plasma of an individual with blood group O? a) Anti-A antibodies b) Anti-B antibodies c) Both anti-A and anti-B antibodies d) No antibodies
n/a	A patient with type A blood has a blood transfusion. The patient develops a fever and chills. What is the most likely cause of this reaction? (A) The patient received incompatible blood. (B) The patient has a viral infection. (C) The patient is allergic to the donor's blood. (D) The patient is experiencing a side effect of the anesthesia.	Which blood group is known as the universal recipient in terms of red blood cell transfusion? a) A positive b) B negative c) O positive d) AB negative
Nerve and Muscle Physiology		
Which of the following is not a neurotransmitter at the neuromuscular junction? a) Acetylcholine b) Dopamine c) Norepinephrine d) GABA	Which of the following is the most important neurotransmitter at the neuromuscular junction? (A) Acetylcholine (B) GABA (C) Glutamate (D) Dopamine	Which of the following is the primary neurotransmitter involved in transmitting signals across the neuromuscular junction? a) Dopamine b) Serotonin c) Acetylcholine d) GABA
Which of the following is not a component of the neuromuscular junction? a) Synaptic vesicles b) Motor end plate c) Axon terminal d) Sarcomere	Which of the following is the most important receptor for acetylcholine at the neuromuscular junction? (A) Nicotinic acetylcholine receptor (B) Muscarinic acetylcholine receptor (C) GABAA receptor (D) Glutamate receptor	Which of the following structures is responsible for releasing acetylcholine into the synaptic cleft at the neuromuscular junction? a) Presynaptic membrane b) Postsynaptic membrane c) Motor end plate d) Sarcomere
Which of the following is not a function of acetylcholine at the neuromuscular junction? a) Depolarization of muscle fiber b) Repolarization of muscle fiber c) Activation of sodium channels d) Inhibition of potassium channels	Which of the following is the most important ion channel involved in the action of acetylcholine at the neuromuscular junction? (A) Sodium channel (B) Potassium channel (C) Chloride channel (D) Calcium channel	The neuromuscular junction is a specialized synapse formed between the motor neuron and the: a) Tendon b) Muscle spindle c) Motor unit d) Golgi tendon organ
Which of the following is not a characteristic feature of the neuromuscular junction? a) Presence of synaptic cleft b) Presence of acetylcholinesterase c) Presence of voltage-gated calcium channels d) Presence of voltage-gated potassium channels	Which of the following is the most important enzyme involved in the breakdown of acetylcholine at the neuromuscular junction? (A) Acetylcholinesterase (B) GABA transaminase (C) Glutamate decarboxylase (D) Dopamine beta-hydroxylase	Which of the following processes is responsible for the termination of the signal transmission at the neuromuscular junction? a) Reuptake of acetylcholine by the presynaptic membrane b) Enzymatic degradation of acetylcholine in the synaptic cleft c) Diffusion of acetylcholine into the bloodstream d) Inhibition of acetylcholine release by the postsynaptic membrane
Which of the following is not a disease associated with the neuromuscular junction? a) Myasthenia gravis b) Lambert-Eaton syndrome c) Botulism d) Parkinson’s disease	Which of the following drugs blocks the action of acetylcholine at the neuromuscular junction? (A) Succinylcholine (B) Curare (C) Neostigmine (D) Edrophonium	Which of the following events occurs first during the activation of the neuromuscular junction? a) Release of calcium ions from the sarcoplasmic reticulum b) Generation of an action potential in the motor neuron c) Binding of acetylcholine to nicotinic receptors on the motor end plate d) Contraction of the muscle fiber
n/a	Which of the following ions is the most important for initiating a muscle contraction? (A) Sodium (B) Potassium (C) Chloride (D) Calcium	Which of the following ions plays a crucial role in initiating skeletal muscle contraction? a) Sodium (Na+) b) Potassium (K+) c) Calcium (Ca2+) d) Chloride (Cl-)
n/a	Which of the following proteins is responsible for the movement of calcium ions into muscle cells? (A) Na,K-ATPase (B) ryanodine receptor (C) troponin C (D) tropomyosin	During skeletal muscle contraction, the release of calcium ions from the sarcoplasmic reticulum is triggered by: a) An increase in ATP levels b) A decrease in pH c) An action potential traveling along the T-tubules d) An influx of sodium ions into the muscle cell
n/a	Which of the following proteins is responsible for binding to calcium ions and exposing the binding sites for actin on myosin? (A) troponin C (B) tropomyosin (C) actin (D) myosin	The binding of calcium ions to troponin during skeletal muscle contraction leads to: a) Activation of myosin ATPase b) Disruption of the cross-bridge formation c) Conformational change in tropomyosin d) Increased permeability of the sarcoplasmic reticulum
n/a	Which of the following proteins is responsible for cross-bridge cycling and muscle contraction? (A) actin (B) myosin (C) troponin C (D) tropomyosin	Which of the following best describes the molecular mechanism underlying the power stroke in skeletal muscle contraction? a) Hydrolysis of ATP by myosin head b) Release of ADP and inorganic phosphate (Pi) from myosin head c) Sliding of actin and myosin filaments past each other d) Dissociation of troponin and tropomyosin complex
n/a	Which of the following factors would decrease the force of a muscle contraction? (A) A decrease in the extracellular calcium concentration (B) A decrease in the number of actin filaments (C) A decrease in the number of myosin filaments (D) A decrease in the amount of ATP available	The removal of calcium ions from the cytosol during skeletal muscle relaxation primarily involves: a) Active transport of calcium ions out of the muscle cell b) Binding of calcium ions to troponin c) Sequestration of calcium ions into the sarcoplasmic reticulum d) Hydrolysis of ATP by actin filaments
Gastrointestinal Physiology		
Which of the following is not a phase of gastric secretion? a) Cephalic phase b) Gastric phase c) Intestinal phase d) Colonic phase	Which of the following is the most important muscle layer in the gastrointestinal tract? (A) Circular muscle layer (B) Longitudinal muscle layer (C) Oblique muscle layer (D) Submucosal muscle layer	Which of the following factors is NOT known to stimulate the release of gastrin, a hormone involved in the regulation of gastrointestinal movements? a) Low pH in the stomach b) Presence of amino acids in the stomach c) Stimulation of the vagus nerve d) Secretion of somatostatin by the stomach
Which of the following is not a function of the stomach? a) Storage of food b) Mechanical digestion of food c) Chemical digestion of food d) Absorption of nutrients	Which of the following types of muscle contraction is responsible for mixing food in the stomach? (A) Peristalsis (B) Segmentation (C) Retrograde peristalsis (D) Pendular movements	Which of the following best describes the role of the migrating motor complex (MMC) in the gastrointestinal tract? a) It promotes the segmentation and mixing of chyme in the small intestine. b) It facilitates the rapid transit of undigested materials through the large intestine. c) It coordinates peristaltic waves in the esophagus during swallowing. d) It sweeps undigested materials from the stomach to the small intestine during fasting.
Which of the following is not a type of motility in the small intestine? a) Segmentation b) Peristalsis c) Migrating motor complex d) Mass movement	Which of the following types of muscle contraction is responsible for moving food from the stomach to the small intestine? (A) Peristalsis (B) Segmentation (C) Retrograde peristalsis (D) Pendular movements	During the cephalic phase of gastric secretion, which of the following stimuli initiates the release of gastric acid and pepsinogen? a) Distension of the stomach wall b) Activation of the sympathetic nervous system c) Stimulation of the gastric mucosa by acetylcholine d) Release of gastrin by the antral G cells
Which of the following is not a function of the large intestine? a) Absorption of water and electrolytes b) Formation and storage of feces c) Digestion of food d) Synthesis of vitamins	Which of the following factors would increase the rate of peristalsis? (A) A decrease in the pH of the stomach contents (B) A decrease in the amount of food in the stomach (C) A decrease in the amount of water in the stomach contents (D) A decrease in the amount of acetylcholine in the stomach	Which of the following hormones is primarily responsible for the relaxation of the lower esophageal sphincter (LES) during swallowing? a) Gastrin b) Secretin c) Cholecystokinin (CCK) d) Vasoactive intestinal peptide (VIP)
Which of the following is not a hormone involved in gastrointestinal motility? a) Gastrin b) Secretin c) Cholecystokinin d) Adrenocorticotropic hormone	Which of the following conditions would decrease the rate of peristalsis? (A) A blockage in the small intestine (B) A decrease in the amount of acetylcholine in the small intestine (C) A decrease in the amount of serotonin in the small intestine (D) A decrease in the amount of nitric oxide in the small intestine	Which of the following factors is responsible for initiating the defecation reflex? a) Distension of the rectum b) Stimulation of the parasympathetic nervous system c) Activation of the external anal sphincter d) Release of motilin by enterochromaffin cells
Which of the following is not a component of saliva? a) Mucin b) Amylase c) Pepsin d) Lysozyme	Which of the following is the most important factor in stimulating gastric acid secretion? (A) Sight and smell of food (B) Distension of the stomach (C) Presence of food in the stomach (D) A decrease in the pH of the stomach contents	Which of the following factors does not influence gastric acid secretion? a) Histamine b) Acetylcholine c) Gastrin d) Somatostatin e) Insulin
Which of the following is not a function of gastric acid secretion? a) Activation of pepsinogen to pepsin b) Denaturation of proteins c) Inhibition of bacterial growth d) Activation of pancreatic enzymes	Which of the following is the most important enzyme in the gastric juice? (A) Pepsin (B) Lipase (C) Trypsin (D) Amylase	Which of the following hormones is responsible for stimulating the release of bile from the gallbladder? a) Gastrin b) Secretin c) Cholecystokinin (CCK) d) Insulin e) Glucagon
Which of the following is not a function of bile secretion? a) Emulsification of fats b) Neutralization of stomach acid c) Absorption of fat-soluble vitamins d) Excretion of bilirubin	Which of the following is the most important function of the gastric juice? (A) To break down proteins into amino acids (B) To break down fats into fatty acids and glycerol (C) To break down carbohydrates into glucose (D) To kill bacteria	Which of the following substances is primarily responsible for the neutralization of gastric acid in the small intestine? a) Sodium bicarbonate b) Pancreatic enzymes c) Gastrin d) Pepsinogen e) Intrinsic factor
Which of the following is not a component of pancreatic juice? a) Amylase b) Lipase c) Trypsinogen d) Pepsin	Which of the following conditions would decrease the secretion of gastric juice? (A) A decrease in the amount of stomach acid (B) A decrease in the amount of food in the stomach (C) A decrease in the amount of gastrin (D) A decrease in the amount of acetylcholine	Which of the following enzymes is responsible for the breakdown of proteins in the stomach? a) Amylase b) Lipase c) Pepsin d) Trypsin e) Chymotrypsin
Which of the following is not a function of intestinal secretions? a) Neutralization of stomach acid b) Digestion and absorption of nutrients c) Lubrication and protection of intestinal mucosa d) Regulation of gastrointestinal motility	Which of the following drugs would decrease the secretion of gastric juice? (A) Omeprazole (B) Pantoprazole (C) Lansoprazole (D) Rabeprazole	Which of the following factors does not play a role in the regulation of intestinal motility? a) Serotonin b) Gastrin c) Motilin d) Cholecystokinin (CCK) e) Vasoactive intestinal peptide (VIP)
Cardiovascular Physiology		
Which of the following is not a mechanism of short-term regulation of blood pressure? a) Baroreceptor reflex b) Chemoreceptor reflex c) Renin-angiotensin-aldosterone system d) Vasopressin secretion	Which of the following ions is the most important for initiating a cardiac action potential? A) Sodium B) Potassium C) Calcium D) Chloride	Which of the following factors primarily regulates blood pressure in the short term? a) Renin-angiotensin-aldosterone system b) Sympathetic nervous system c) Atrial natriuretic peptide d) Endothelin-1
Which of the following is not a function of angiotensin II? a) Vasoconstriction b) Sodium and water retention c) Potassium excretion d) Stimulation of aldosterone secretion	Which of the following channels is responsible for the rapid influx of sodium ions during the initial phase of the cardiac action potential? a) Sodium channels b) Potassium channels c) Calcium channels d) Chloride channels	Which of the following hormones acts directly on the kidneys to increase sodium reabsorption and subsequently raise blood pressure? a) Aldosterone b) Antidiuretic hormone (ADH) c) Atrial natriuretic peptide d) Parathyroid hormone
Which of the following is not a function of atrial natriuretic peptide? a) Vasodilation b) Sodium and water excretion c) Potassium retention d) Inhibition of renin secretion	Which of the following channels is responsible for the slow inward flow of calcium ions during the plateau phase of the cardiac action potential? a) Sodium channels b) Potassium channels c) Calcium channels d) Chloride channels	Which of the following structures is responsible for the long-term regulation of blood pressure through the secretion of renin? a) Juxtaglomerular apparatus b) Renal tubules c) Adrenal cortex d) Pituitary gland
Which of the following is not a function of nitric oxide? a) Vasodilation b) Inhibition of platelet aggregation c) Stimulation of renin secretion d) Inhibition of smooth muscle proliferation	Which of the following channels is responsible for the repolarization phase of the cardiac action potential? a) Sodium channels b) Potassium channels c) Calcium channels d) Chloride channels	Which of the following is a potential mechanism for the regulation of blood pressure by baroreceptors? a) Decreased sympathetic stimulation of blood vessels b) Increased renin release from the kidneys c) Direct activation of the parasympathetic nervous system d) Inhibition of the renin-angiotensin-aldosterone system
Which of the following is not a function of endothelin? a) Vasoconstriction b) Stimulation of aldosterone secretion c) Inhibition of nitric oxide synthesis d) Stimulation of smooth muscle proliferation	Which of the following drugs blocks the sodium channels in cardiac muscle cells? a) Lidocaine b) Procainamide c) Quinidine d) Verapamil	Which of the following statements accurately describes the effect of nitric oxide on blood pressure regulation? a) Nitric oxide causes vasoconstriction, leading to increased blood pressure. b) Nitric oxide inhibits the production of angiotensin II, resulting in decreased blood pressure. c) Nitric oxide stimulates the release of aldosterone, leading to increased blood pressure. d) Nitric oxide causes vasodilation, resulting in decreased blood pressure.
Which of the following is not a phase of the cardiac action potential? a) Resting phase b) Depolarization phase c) Plateau phase d) Repolarization phase	Which of the following is the most important factor in regulating blood pressure? (A) Cardiac output (B) Peripheral vascular resistance (C) Blood volume (D) Blood viscosity	Which ion plays a crucial role in the initiation and propagation of cardiac impulses? a) Sodium (Na+) b) Potassium (K+) c) Calcium (Ca2+) d) Chloride (Cl-)
Which of the following is not a function of the sinoatrial node? a) Generation of action potentials b) Conduction of action potentials c) Modulation of heart rate d) Regulation of blood pressure	Which of the following hormones is the most important in increasing blood pressure? (A) Angiotensin II (B) Epinephrine (C) Norepinephrine (D) Aldosterone	Which ion is primarily responsible for the depolarization phase of the cardiac action potential? a) Sodium (Na+) b) Potassium (K+) c) Calcium (Ca2+) d) Chloride (Cl-)
Which of the following is not a function of the atrioventricular node? a) Delay of impulse conduction b) Modulation of heart rate c) Conduction of action potentials d) Regulation of blood pressure	Which of the following drugs is the most effective in lowering blood pressure? (A) Angiotensin-converting enzyme (ACE) inhibitors (B) Calcium channel blockers (C) Beta-blockers (D) Diuretics	During the resting state of a cardiac cell, the concentration of which ion is higher outside the cell compared to inside? a) Sodium (Na+) b) Potassium (K+) c) Calcium (Ca2+) d) Chloride (Cl-)
Which of the following is not a function of the bundle of His? a) Conduction of action potentials to the ventricles b) Modulation of heart rate c) Regulation of blood pressure d) Separation of atrial and ventricular contractions	Which of the following conditions is associated with high blood pressure? (A) Obesity (B) Diabetes mellitus (C) Kidney disease (D) All of the above	The movement of which ion across the cell membrane leads to the repolarization phase of the cardiac action potential? a) Sodium (Na+) b) Potassium (K+) c) Calcium (Ca2+) d) Chloride (Cl-)
Which of the following is not a function of the Purkinje fibers? a) Conduction of action potentials to the ventricles b) Modulation of heart rate c) Regulation of blood pressure d) Coordination of ventricular contractions	Which of the following lifestyle changes is the most effective in preventing high blood pressure? (A) Losing weight (B) Eating a healthy diet (C) Exercising regularly (D) All of the above	Which ion channels are responsible for the initial rapid depolarization of the cardiac action potential? a) Sodium (Na+) channels b) Potassium (K+) channels c) Calcium (Ca2+) channels d) Chloride (Cl-) channels
Respiratory Physiology		
Which of the following is not a physiological response to high altitude? a) Hyperventilation b) Increased heart rate c) Increased red blood cell production d) Decreased blood pressure	Which of the following is the most important factor in acclimatization to high altitude? A) Increased red blood cell production B) Increased breathing rate C) Increased heart rate D) Decreased blood pressure	Which of the following physiological adaptations occurs in response to high altitude exposure? a) Increased red blood cell production b) Decreased heart rate c) Decreased breathing rate d) Decreased blood pressure
Which of the following is not a physiological response to deep sea diving? a) Increased heart rate b) Increased blood pressure c) Increased oxygen consumption d) Decreased carbon dioxide production	Which of the following conditions is most likely to occur at high altitudes? A) Acute mountain sickness B) High-altitude pulmonary edema C) High-altitude cerebral edema D) All of the above	During deep sea diving, which of the following gases poses the greatest risk to divers? a) Nitrogen b) Oxygen c) Carbon dioxide d) Helium
Which of the following is not a symptom of acute mountain sickness? a) Headache b) Nausea and vomiting c) Shortness of breath d) Hypertension	Which of the following drugs is used to treat acute mountain sickness? A) Acetazolamide B) Dexamethasone C) Nifedipine D) All of the above	Which of the following is a common physiological response to high altitude exposure? a) Increased urine production b) Decreased blood viscosity c) Increased oxygen saturation in arterial blood d) Decreased respiratory rate
Which of the following is not a symptom of decompression sickness? a) Joint pain b) Skin rash c) Dizziness and confusion d) Paralysis	Which of the following is the most important factor in preventing decompression sickness? A) Avoiding rapid ascent B) Adequate hydration C) Avoiding alcohol and caffeine D) All of the above	What is the primary mechanism by which the body adapts to decreased oxygen availability at high altitudes? a) Increased production of hemoglobin b) Increased production of carbon dioxide c) Decreased oxygen consumption by tissues d) Decreased ventilation rate
Which of the following is not a mechanism of oxygen toxicity? a) Inactivation of enzymes b) Formation of free radicals c) Damage to cell membranes d) Stimulation of erythropoietin secretion	Which of the following conditions is most likely to occur during deep sea diving? A) Decompression sickness B) Nitrogen narcosis C) Oxygen toxicity D) All of the above	Which of the following physiological changes occurs during deep sea diving? a) Decreased heart rate b) Increased lung compliance c) Increased blood volume d) Increased oxygen saturation in venous blood
Which of the following is not a function of hemoglobin? a) Transport of oxygen b) Transport of carbon dioxide c) Buffering of hydrogen ions d) Regulation of blood pressure	Which of the following is the most important factor in the transport of oxygen from the lungs to the tissues? A) The partial pressure gradient of oxygen B) The concentration of hemoglobin in the blood C) The affinity of hemoglobin for oxygen D) The cardiac output	Which of the following factors primarily determines the rate of diffusion of respiratory gases across the alveolar-capillary membrane? a) Thickness of the respiratory membrane b) Concentration gradient of the gases c) Surface area available for diffusion d) Solubility of the gases in blood
Which of the following is not a factor affecting the oxygen-hemoglobin dissociation curve? a) pH b)Temperature c) Carbon dioxide concentration d) Blood pressure	Which of the following factors would increase the affinity of hemoglobin for oxygen? A) A decrease in the pH of the blood B) A decrease in the temperature of the blood C) A decrease in the concentration of carbon dioxide in the blood D) All of the above	In which form is the majority of carbon dioxide transported from the tissues to the lungs? a) Dissolved in plasma b) As bicarbonate ions c) Bound to hemoglobin d) Attached to red blood cells
Which of the following is not a mechanism of carbon dioxide transport? a) Dissolved in plasma b) Bound to hemoglobin c) As bicarbonate ions d) As carbamino compounds	Which of the following factors would decrease the affinity of hemoglobin for oxygen? A) An increase in the pH of the blood B) An increase in the temperature of the blood C) An increase in the concentration of carbon dioxide in the blood D) All of the above	Which of the following factors increases the affinity of hemoglobin for oxygen? a) Decreased pH (Bohr effect) b) Increased temperature c) Decreased carbon dioxide levels d) Increased 2,3-bisphosphoglycerate (BPG) levels
Which of the following is not a mechanism of oxygen transport? a) Dissolved in plasma b) Bound to hemoglobin c) As bicarbonate ions d) As carbamino compounds	Which of the following is the most important factor in the transport of carbon dioxide from the tissues to the lungs? A) The partial pressure gradient of carbon dioxide B) The concentration of hemoglobin in the blood C) The affinity of hemoglobin for carbon dioxide D) The cardiac output	Which of the following conditions would result in a leftward shift of the oxygen-hemoglobin dissociation curve? a) Increased pH b) Decreased temperature c) Decreased partial pressure of carbon dioxide d) Decreased levels of 2,3-bisphosphoglycerate (BPG)
Which of the following is not a factor affecting the carbon dioxide-hemoglobin dissociation curve? a) pH b) Temperature c) Oxygen concentration d) Blood pressure	Which of the following drugs would increase the amount of carbon dioxide that is carried by the blood? A) Carbonic anhydrase inhibitors B) Alkalinizing agents C) Hyperbaric oxygen therapy D) All of the above	Which of the following is the primary mode of oxygen transport in the blood? a) Dissolved in plasma b) Bound to hemoglobin c) Combined with carbon dioxide as carbaminohemoglobin d) Attached to red blood cells
Renal Physiology		
Which of the following is not a component of the filtration barrier in the glomerulus? a) Endothelial cells b) Basement membrane c) Podocytes d) Mesangial cells	Which of the following is the most important factor in determining the glomerular filtration rate (GFR)? A) The hydrostatic pressure in the glomerular capillaries B) The oncotic pressure in the glomerular capillaries C) The filtration fraction D) All of the above	Which of the following factors does not directly affect the glomerular filtration rate (GFR)? a) Blood pressure b) Afferent arteriole constriction c) Efferent arteriole dilation d) Plasma protein concentration
Which of the following is not a factor affecting the glomerular filtration rate? a) Blood pressure b) Renal blood flow c) Plasma protein concentration d) Sympathetic nervous system activity	Which of the following factors would increase the GFR? A) An increase in the hydrostatic pressure in the glomerular capillaries B) A decrease in the oncotic pressure in the glomerular capillaries C) An increase in the filtration fraction D) All of the above	Which of the following statements about glomerular filtration rate (GFR) is true? a) GFR increases when there is constriction of the afferent arteriole. b) GFR increases when there is dilation of the efferent arteriole. c) GFR decreases when there is an increase in the plasma protein concentration. d) GFR is not affected by changes in blood pressure.
Which of the following is not a function of angiotensin II? a) Vasoconstriction b) Sodium and water retention c) Potassium excretion d) Stimulation of aldosterone secretion	Which of the following factors would decrease the GFR? A) A decrease in the hydrostatic pressure in the glomerular capillaries B) An increase in the oncotic pressure in the glomerular capillaries C) A decrease in the filtration fraction D) All of the above	Which of the following substances is not freely filtered at the glomerulus? a) Water b) Glucose c) Sodium d) Urea
Which of the following is not a function of atrial natriuretic peptide? a) Vasodilation b) Sodium and water excretion c) Potassium retention d) Inhibition of renin secretion	Which of the following conditions would be associated with a decreased GFR? A) Diabetes mellitus B) Hypertension C) Glomerulonephritis D) All of the above	Which of the following hormones plays a role in the regulation of glomerular filtration rate (GFR)? a) Insulin b) Epinephrine c) Aldosterone d) Growth hormone
Which of the following is not a function of antidiuretic hormone? a) Water reabsorption in the collecting ducts b) Vasoconstriction c) Stimulation of aldosterone secretion d) Inhibition of urine production	Which of the following drugs would increase the GFR? A) ACE inhibitors B) Calcium channel blockers C) Diuretics D) All of the above	Which of the following mechanisms contributes to the maintenance of a constant glomerular filtration rate (GFR) despite changes in blood pressure? a) Myogenic regulation b) Tubuloglomerular feedback c) Renin-angiotensin-aldosterone system d) Sympathetic nervous system activation
Which of the following is not a mechanism of acid-base regulation by the kidneys? a) Reabsorption of bicarbonate ions b) Secretion of hydrogen ions c) Formation of ammonium ions d) Formation of carbonic acid	Which of the following is the most important buffer system in the blood? A) Carbonic acid-bicarbonate buffer system B) Phosphate buffer system C) Protein buffer system D) All of the above	Which of the following mechanisms primarily contributes to the kidney's ability to regulate acid-base balance? a) Reabsorption of bicarbonate ions b) Secretion of hydrogen ions c) Formation of ammonia d) Production of carbonic anhydrase
Which of the following is not a buffer system involved in acid-base balance? a) Bicarbonate buffer system b) Phosphate buffer system c) Protein buffer system d) Chloride buffer system	Which of the following is the most important way that the kidneys regulate acid-base balance? A) Excretion of hydrogen ions B) Excretion of bicarbonate ions C) Reabsorption of bicarbonate ions D) All of the above	In response to acidosis, how does the kidney compensate to restore acid-base balance? a) Increase secretion of bicarbonate ions b) Increase reabsorption of hydrogen ions c) Decrease production of carbonic anhydrase d) Decrease formation of ammonia
Which of the following is not a cause of metabolic acidosis? a) Diarrhea b) Renal failure c) Hyperventilation d) Lactic acidosis	Which of the following conditions would cause the kidneys to retain hydrogen ions? A) Metabolic acidosis B) Respiratory alkalosis C) Metabolic alkalosis D) Respiratory acidosis	Which of the following is a key role of the proximal tubule in acid-base balance? a) Reabsorption of filtered bicarbonate ions b) Secretion of ammonium ions c) Reabsorption of hydrogen ions d) Secretion of carbonic anhydrase
Which of the following is not a cause of metabolic alkalosis? a) Vomiting b) Hypokalemia c) Diuretic use d) Respiratory failure	Which of the following conditions would cause the kidneys to excrete bicarbonate ions? A) Metabolic alkalosis B) Respiratory acidosis C) Metabolic acidosis D) Respiratory alkalosis	In a patient with respiratory alkalosis, which renal response would be expected? a) Increased reabsorption of bicarbonate ions b) Increased secretion of hydrogen ions c) Decreased reabsorption of bicarbonate ions d) Decreased secretion of ammonium ions
Which of the following is not a cause of respiratory acidosis? a) Chronic obstructive pulmonary disease (COPD) b) Pulmonary embolism c) Hyperventilation d) Opioid overdose	Which of the following drugs would increase the excretion of hydrogen ions by the kidneys? A) Acetazolamide B) Furosemide C) Hydrochlorothiazide D) All of the above	Which of the following is a consequence of metabolic acidosis on renal function? a) Increased excretion of bicarbonate ions b) Decreased secretion of hydrogen ions c) Decreased reabsorption of ammonium ions d) Increased production of carbonic anhydrase
Endocrine Physiology		
Which of the following is not a function of growth hormone? a) Stimulation of protein synthesis b) Stimulation of lipolysis c) Stimulation of glucose uptake d) Inhibition of insulin secretion	Which of the following is the most important function of growth hormone? A) Stimulate cell growth and division B) Promote protein synthesis C) Increase bone growth D) All of the above	Which of the following statements regarding growth hormone (GH) is true? a) GH is released from the posterior pituitary gland. b) GH promotes growth in long bones through direct stimulation of osteoblasts. c) GH acts primarily on the thyroid gland to regulate metabolism. d) GH levels are highest during adolescence and decline with age.
Which of the following is not a factor affecting growth hormone secretion? a) Sleep b) Exercise c) Stress d) Blood pressure	Which of the following cells produce growth hormone? A) Somatotrophs B) Gonadotrophs C) Thyrotrophs D) All of the above	Which of the following conditions is associated with excessive secretion of growth hormone in adulthood? a) Gigantism b) Acromegaly c) Cushing's syndrome d) Hypothyroidism
Which of the following is not a cause of growth hormone deficiency? a) Pituitary tumor b) Trauma to the head c) Radiation therapy d) Hyperthyroidism	Which of the following factors stimulates the release of growth hormone? A) Sleep B) Exercise C) Stress D) All of the above	Growth hormone deficiency in children can result in which of the following conditions? a) Cushing's syndrome b) Hyperthyroidism c) Achondroplasia d) Dwarfism
Which of the following is not a cause of acromegaly? a) Pituitary tumor b) Hyperthyroidism c) Adrenal gland tumor d) Carcinoid tumor	Which of the following factors inhibits the release of growth hormone? A) High blood sugar levels B) High levels of insulin C) High levels of thyroid hormone D) All of the above	Which of the following organs is the primary target of growth hormone action? a) Liver b) Thyroid gland c) Pancreas d) Adrenal gland
Which of the following is not a symptom of gigantism? a) Increased height b) Enlarged hands and feet c) Coarsening of facial features d) Hypoglycemia	Which of the following conditions is associated with a deficiency of growth hormone? A) Prader-Willi syndrome B) Turner syndrome C) Noonan syndrome D) All of the above	Which of the following factors stimulates the secretion of growth hormone? a) High blood glucose levels b) Low blood glucose levels c) High levels of thyroid hormones d) High levels of cortisol
Which of the following is not a function of thyroid hormone? a) Stimulation of protein synthesis b) Stimulation of lipolysis c) Stimulation of glucose uptake d) Inhibition of insulin secretion	Which of the following is the most important function of thyroid hormone? A) Regulate metabolism B) Promote growth and development C) Increase heart rate and blood pressure D) All of the above	Which of the following hormones is primarily responsible for the negative feedback regulation of thyroid-stimulating hormone (TSH) secretion? a) Thyroxine (T4) b) Triiodothyronine (T3) c) Thyroid-releasing hormone (TRH) d) Thyroid-stimulating hormone receptor (TSH-R)
Which of the following is not a factor affecting thyroid hormone secretion? a) Iodine intake b) TSH secretion c) Stress d) Blood pressure	Which of the following cells produce thyroid hormone? A) Thyroid follicular cells B) Somatotrophs C) Thyrotrophs D) All of the above	Which of the following statements is true regarding the transport of thyroid hormones in the blood? a) The majority of thyroid hormones in the blood are bound to thyroid-binding globulin (TBG). b) Free thyroxine (FT4) is the biologically active form of thyroid hormone. c) The uptake of thyroid hormones by target cells occurs via passive diffusion. d) Thyroid hormones are primarily transported in the blood as free, unbound molecules.
Which of the following is not a cause of hypothyroidism? a) Hashimoto’s thyroiditis b) Iodine deficiency c) Pituitary tumor d) Radiation therapy	Which of the following factors stimulates the release of thyroid hormone? A) Thyrotropin-releasing hormone (TRH) B) Thyroid-stimulating hormone (TSH) C) Calcitonin D) All of the above	Which of the following enzymes is responsible for the conversion of thyroxine (T4) to triiodothyronine (T3) in target tissues? a) Thyroid peroxidase (TPO) b) Thyroxine-binding globulin (TBG) c) Iodothyronine deiodinase (ID) d) Thyroid-stimulating hormone (TSH)
Which of the following is not a cause of hyperthyroidism? a) Graves’ disease b) Thyroiditis c) Pituitary tumor d) Thyroid nodules	Which of the following factors inhibits the release of thyroid hormone? A) High levels of thyroid hormone B) Low levels of calcium C) High levels of cortisol D) All of the above	Which of the following conditions is characterized by low levels of thyroid-stimulating hormone (TSH) and elevated levels of free thyroxine (FT4)? a) Hyperthyroidism b) Hypothyroidism c) Graves' disease d) Hashimoto's thyroiditis
Which of the following is not a symptom of hypothyroidism? a) Fatigue b) Weight gain c) Cold intolerance d) Hyperactivity	Which of the following conditions is associated with an excess of thyroid hormone? A) Graves' disease B) Hashimoto's thyroiditis C) Thyroid cancer D) All of the above	Which of the following physiological processes is NOT directly influenced by thyroid hormones? a) Metabolic rate b) Growth and development c) Regulation of blood pressure d) Calcium homeostasis
Reproductive Physiology		
Which of the following is not a hormone involved in parturition? a) Oxytocin b) Progesterone c) Estrogen d) Relaxin	Which of the following is the most effective method of contraception? A) Vasectomy B) Tubal ligation C) Oral contraceptive pills D) All of the above	Which of the following hormones is responsible for initiating and maintaining contractions during labor and parturition? a) Estrogen b) Progesterone c) Oxytocin d) Prolactin
Which of the following is not a stage of parturition? a) Dilation b) Expulsion c) Placental d) Preparatory	Which of the following methods of contraception is the most reversible? A) Condoms B) Diaphragm C) Intrauterine device (IUD) D) All of the above	Which of the following physiological changes occurs during parturition? a) Decreased blood pressure b) Decreased uterine contractions c) Rupture of amniotic sac d) Decreased production of prostaglandins
Which of the following is not a function of prolactin? a) Milk production b) Milk ejection c) Inhibition of ovulation d) Stimulation of uterine contractions	Which of the following methods of contraception is most likely to cause side effects? A) Oral contraceptive pills B) Intramuscular injection C) Implant D) All of the above	During lactation, what is the primary hormone responsible for milk synthesis and secretion? a) Oxytocin b) Progesterone c) Estrogen d) Prolactin
Which of the following is not a function of oxytocin? a) Milk ejection b) Uterine contractions c) Inhibition of ovulation d) Bonding between mother and infant	Which of the following methods of contraception is most likely to fail? A) Withdrawal B) Rhythm method C) Coitus interruptus D) All of the above	Which of the following is a primary mechanism by which breast milk is produced and regulated during lactation? a) Prolactin stimulates milk ejection b) Oxytocin stimulates milk production c) Prolactin inhibits milk production d) Oxytocin inhibits milk ejection
Which of the following is not a cause of lactation failure? a) Insufficient glandular tissue b) Hormonal imbalance c) Breastfeeding too frequently d) Medications	Which of the following methods of contraception is most likely to be used by adolescents? A) Condoms B) Oral contraceptive pill C) Diaphragm D) All of the above	Which of the following events triggers the let-down reflex during breastfeeding? a) Increase in maternal blood pressure b) Suckling of the infant c) Decrease in maternal blood sugar levels d) Administration of oxytocin medication
Which of the following is not a type of hormonal contraception? a) Combined oral contraceptive pill b) Progestin-only pill c) Intrauterine device (IUD) d) Condom	Which of the following is the most important factor in the initiation of labor? A) The fetus reaching a certain gestational age B) The release of oxytocin from the posterior pituitary gland C) The stretching of the cervix and vagina D) All of the above	Which of the following is NOT a mechanism of action for hormonal contraception? a) Inhibition of ovulation b) Thickening of cervical mucus c) Alteration of endometrial lining d) Inhibition of sperm motility
Which of the following is not a mechanism of action of hormonal contraception? a) Inhibition of ovulation b) Thickening of cervical mucus c) Inhibition of implantation d) Stimulation of uterine contractions	Which of the following hormones is responsible for stimulating the production of milk? A) Prolactin B) Oxytocin C) Estrogen D) All of the above	Which of the following contraceptive methods provides the highest efficacy rate? a) Condoms b) Oral contraceptives c) Intrauterine devices (IUDs) d) Fertility awareness-based methods
Which of the following is not a type of emergency contraception? a) Levonorgestrel pill b) Ulipristal acetate pill c) Copper IUD d) Combined oral contraceptive pill	Which of the following factors is important for the milk to be ejected from the breast? A) The release of oxytocin from the posterior pituitary gland B) The sucking of the baby C) The stretching of the breasts D) All of the above	Which of the following is a side effect associated with the use of combined oral contraceptives? a) Weight gain b) Decreased libido c) Increased risk of breast cancer d) Decreased risk of ectopic pregnancy
Which of the following is not a cause of contraceptive failure? a) Incorrect use b) Hormonal imbalance c) Medications d) Condom breakage	Which of the following conditions can interfere with lactation? A) Premature birth B) Hypothyroidism C) Stress D) All of the above	Which hormone is primarily responsible for the maintenance of pregnancy and inhibition of ovulation during the menstrual cycle? a) Follicle-stimulating hormone (FSH) b) Luteinizing hormone (LH) c) Estrogen d) Progesterone
Which of the following is not a benefit of hormonal contraception? a) Reduced risk of ovarian cancer b) Reduced risk of endometrial cancer c) Reduced risk of sexually transmitted infections (STIs) d) Reduced menstrual cramps	Which of the following is the best way to prevent mastitis? A) Frequent breastfeeding B) Proper hygiene C) Avoiding tight clothing D) All of the above	Which of the following is an example of a barrier method of contraception? a) Oral contraceptive pills b) Contraceptive patch c) Diaphragm d) Implantable rod
Neurophysiology		
Which of the following is not a division of the autonomic nervous system? a) Sympathetic b) Parasympathetic c) Somatic d) Enteric	Which of the following is the most important function of the autonomic nervous system? A) Regulate involuntary functions of the body B) Control heart rate and blood pressure C) Stimulate digestion and elimination D) All of the above	Which of the following statements accurately describes the effect of the parasympathetic nervous system on the heart rate? a) Parasympathetic stimulation increases heart rate. b) Parasympathetic stimulation decreases heart rate. c) Parasympathetic stimulation has no effect on heart rate. d) Parasympathetic stimulation causes irregular heart rate.
Which of the following is not a function of the sympathetic nervous system? a) Increased heart rate b) Bronchoconstriction c) Pupil dilation d) Increased blood pressure	Which of the following divisions of the autonomic nervous system is responsible for the "fight-or-flight" response? A) Sympathetic nervous system B) Parasympathetic nervous system C) Enteric nervous system D) All of the above	During sympathetic activation, which of the following changes occur in the airways? a) Constriction of bronchial smooth muscles. b) Relaxation of bronchial smooth muscles. c) No change in bronchial smooth muscle tone. d) Increased production of mucus in the airways
Which of the following is not a function of the parasympathetic nervous system? a) Decreased heart rate b) Bronchodilation c) Pupil constriction d) Increased blood pressure	Which of the following neurotransmitters is released by the sympathetic nervous system? A) Epinephrine B) Norepinephrine C) Acetylcholine D) All of the above	Which neurotransmitter is primarily responsible for transmission at the ganglionic synapses of the sympathetic nervous system? a) Acetylcholine (ACh) b) Norepinephrine (NE) c) Dopamine d) Serotonin
Which of the following is not a neurotransmitter involved in the autonomic nervous system? a) Acetylcholine b) Norepinephrine c) Dopamine d) Epinephrine	Which of the following neurotransmitters is released by the parasympathetic nervous system? A) Acetylcholine B) Dopamine C) GABA D) All of the above	The baroreceptor reflex is an important mechanism for regulating: a) Blood glucose levels b) Blood pressure c) Body temperature d) Respiratory rate
Which of the following is not a cause of autonomic dysfunction? a) Diabetes mellitus b) Parkinson’s disease c) Multiple sclerosis d) Hypertension	Which of the following conditions is associated with an overactive sympathetic nervous system? A) Anxiety B) Hyperthyroidism C) Heart disease D) All of the above	Which of the following statements regarding the autonomic innervation of the gastrointestinal tract is correct? a) Parasympathetic stimulation increases gastrointestinal motility. b) Sympathetic stimulation increases gastrointestinal motility. c) Parasympathetic stimulation decreases gastrointestinal motility. d) Sympathetic stimulation has no effect on gastrointestinal motility.
Which of the following is not a layer of the retina? a) Photoreceptor layer b) Pigment epithelium layer c) Ganglion cell layer d) Sclera	Which of the following is the most important function of the retina? A) Convert light into electrical signals B) Transmit electrical signals to the brain C) Process electrical signals into images D) All of the above	Which of the following statements accurately describes the process of accommodation in the physiology of vision? a) The ciliary muscles relax, causing the lens to flatten and focus on distant objects. b) The ciliary muscles contract, causing the lens to thicken and focus on near objects. c) The iris constricts, allowing more light to enter the eye and improve near vision. d) The retina adjusts its sensitivity to different wavelengths of light for optimal visual perception.
Which of the following is not a type of photoreceptor? a) Rods b) Cones c) Bipolar cells d) Horizontal cells	Which of the following cells in the retina are responsible for converting light into electrical signals? A) Rods B) Cones C) Bipolar cells D) All of the above	In the physiology of vision, which structure is responsible for detecting and converting light into electrical signals that can be interpreted by the brain? a) Cornea b) Lens c) Retina d) Optic nerve
Which of the following is not a function of the iris? a) Regulation of pupil size b) Regulation of light entering the eye c) Production of aqueous humor d) Protection of the eye from bright light	Which of the following cells in the retina are responsible for transmitting electrical signals to the brain? A) Rods B) Cones C) Bipolar cells D) Ganglion cells	Which of the following visual pathways is responsible for transmitting information from the left visual field to the right hemisphere of the brain? a) Optic nerve b) Optic chiasm c) Optic tract d) Lateral geniculate nucleus
Which of the following is not a cause of visual impairment? a) Cataracts b) Glaucoma c) Macular degeneration d) Astigmatism	Which of the following cells in the retina are responsible for processing electrical signals into images? A) Ganglion cells B) Bipolar cells C) Amacrine cells D) Horizontal cells	The perception of color in the physiology of vision is primarily mediated by specialized photoreceptor cells in the retina known as a) Rods b) Cones c) Bipolar cells d) Ganglion cells
Which of the following is not a function of the optic nerve? a) Transmission of visual information to the brain b) Regulation of pupil size c) Coordination of eye movements d) Perception of color	Which of the following conditions is associated with damage to the retina? A) Cataract B) Glaucoma C) Age-related macular degeneration D) All of the above	Which of the following statements accurately describes the function of the fovea in the physiology of vision? a) It is responsible for peripheral vision and detecting motion. b) It contains a high density of cones, providing detailed central vision. c) It helps to control the amount of light entering the eye through the pupil. d) It is the area where the optic nerve exits the eye, transmitting visual information to the brain.
Integrated Physiology		
Which of the following is not a respiratory response to exercise? a) Increased tidal volume b) Increased respiratory rate c) Decreased alveolar ventilation d) Increased oxygen uptake	Which of the following is the most important factor that limits exercise performance? A) Heart rate B) Blood pressure C) Oxygen delivery to the muscles D) All of the above	Which of the following factors contributes to increased cardiac output during exercise? a) Decreased sympathetic nervous system activity b) Increased systemic vascular resistance c) Decreased heart rate d) Increased stroke volume
Which of the following is not a cardiovascular response to exercise? a) Increased heart rate b) Increased stroke volume c) Decreased cardiac output d) Increased blood pressure	Which of the following hormones is responsible for increasing heart rate during exercise? A) Epinephrine B) Norepinephrine C) Thyroid hormone D) All of the above	During moderate-intensity exercise, which of the following metabolic adjustments is most likely to occur? a) Decreased utilization of fatty acids as an energy source b) Increased production of lactate in skeletal muscles c) Decreased reliance on glucose as a primary fuel d) Increased oxygen delivery to adipose tissue
Which of the following is not a metabolic response to exercise? a) Increased glycogenolysis b) Increased lipolysis c) Decreased insulin secretion d) Increased protein synthesis	Which of the following factors is most important for increasing oxygen delivery to the muscles during exercise? A) Increased heart rate B) Increased stroke volume C) Increased red blood cell count D) All of the above	Which of the following best describes the primary mechanism responsible for respiratory adjustments during exercise? a) Decreased respiratory rate to conserve energy b) Increased alveolar ventilation to maintain oxygen and carbon dioxide levels c) Constriction of bronchioles to increase airway resistance d) Decreased tidal volume to reduce oxygen consumption
Which of the following is not a factor affecting maximal oxygen uptake? a) Age b) Sex c) Body weight d) Blood type	Which of the following metabolic pathways is most important for providing energy during exercise? A) Aerobic glycolysis B) Anaerobic glycolysis C) Oxidative phosphorylation D) All of the above	During high-intensity exercise, which of the following statements regarding oxygen consumption is correct? a) Oxygen consumption decreases due to increased anaerobic metabolism b) Oxygen consumption remains constant due to a plateau effect c) Oxygen consumption increases linearly with exercise intensity d) Oxygen consumption decreases due to decreased cardiac output
Which of the following is not a benefit of regular exercise? a) Improved cardiovascular health b) Improved respiratory health c) Reduced risk of type 2 diabetes d) Reduced risk of osteoporosis	Which of the following is the most important factor that determines how long an individual can exercise? A) The amount of oxygen that can be delivered to the muscles B) The amount of energy that can be produced by the muscles C) The ability of the muscles to remove waste products D) All of the above	Which of the following adaptations is commonly observed in individuals who engage in regular aerobic exercise? a) Increased resting heart rate b) Decreased blood volume c) Increased maximal oxygen uptake (VO2 max) d) Decreased capillary density in skeletal muscles
Which of the following is not a mechanism of heat loss? a) Radiation b) Conduction c) Convection d) Evaporation	Which of the following is the most important factor that regulates body temperature? A) The hypothalamus B) The thyroid gland C) The adrenal glands D) All of the above	Which of the following physiological mechanisms is primarily responsible for maintaining core body temperature during exposure to cold environments? a) Shivering thermogenesis b) Vasodilation c) Sweating d) Piloerection
Which of the following is not a mechanism of heat production? a) Metabolism b) Shivering c) Sweating d) Hormonal regulation	Which of the following mechanisms is responsible for heat loss from the body? A) Radiation B) Conduction C) Convection D) Evaporation	In a fever response, which of the following hypothalamic centers is primarily responsible for initiating the increase in body temperature? a) Preoptic anterior hypothalamus (POAH) b) Suprachiasmatic nucleus (SCN) c) Arcuate nucleus d) Ventromedial nucleus
Which of the following is not a thermoregulatory center in the brain? a) Hypothalamus b) Thalamus c) Cerebellum d) Medulla oblongata	Which of the following mechanisms is responsible for heat gain from the body? A) Radiation B) Conduction C) Convection D) Metabolism	Which of the following statements best describes the role of brown adipose tissue (BAT) in temperature regulation? a) BAT is responsible for heat dissipation through vasodilation. b) BAT produces heat through non-shivering thermogenesis. c) BAT triggers peripheral vasoconstriction to conserve heat. d) BAT promotes sweating and evaporative cooling.
Which of the following is not a cause of hypothermia? a) Prolonged exposure to cold b) Alcohol consumption c) Dehydration d) Hypoglycemia	Which of the following conditions is associated with an increased risk of hypothermia? A) Aging B) Alcohol intoxication C) Malnutrition D) All of the above	Which of the following peripheral thermoreceptors is responsible for detecting cold temperatures and transmitting signals to the central thermoregulatory centers? a) Meissner's corpuscles b) Pacinian corpuscles c) Ruffini endings d) Krause end bulbs
Which of the following is not a cause of hyperthermia? a) Prolonged exposure to heat b) Exercise c) Infection d) Hypoglycemia	Which of the following conditions is associated with an increased risk of hyperthermia? A) Strenuous exercise B) Heat stroke C) Dehydration D) All of the above	During heat stroke, which of the following physiological responses is impaired? a) Vasodilation of cutaneous blood vessels b) Increased sweating and evaporative cooling c) Activation of the sympathetic nervous system d) Shivering thermogenesis

## References

[1] Wang P (2019). On defining artificial intelligence. J Artif Gen Intell.

[2] Fatani B (2023). ChatGPT for future medical and dental research. Cureus.

[3] Sharma M, Savage C, Nair M, Larsson I, Svedberg P, Nygren JM (2022). Artificial intelligence applications in health care practice: Scoping review. J Med Internet Res.

[4] Paranjape K, Schinkel M, Nannan Panday R, Car J, Nanayakkara P (2019). Introducing artificial intelligence training in medical education. JMIR Med Educ.

[5] Zhao J, Wu M, Zhou L, Wang X, Jia J (2022). Cognitive psychology-based artificial intelligence review. Front Neurosci.

[6] Joyner MJ (2011). Why physiology matters in medicine. Physiology (Bethesda).

[7] Zaidi NL, Grob KL, Monrad SM (2018). Pushing critical thinking skills with multiple-choice questions: Does Bloom's taxonomy work?. Acad Med.

[8] Das D, Kumar N, Longjam LA, Sinha R, Deb Roy A, Mondal H, Gupta P (2023). Assessing the capability of ChatGPT in answering first- and second-order knowledge questions on microbiology as per competency-based medical education curriculum. Cureus.

[9] Sinha RK, Deb Roy A, Kumar N, Mondal H (2023). Applicability of ChatGPT in assisting to solve higher order problems in pathology. Cureus.

[10] Ghosh A, Bir A (2023). Evaluating ChatGPT’s ability to solve higher-order questions on the competency-based medical education curriculum in medical biochemistry. Cureus.

[11] Kung TH, Cheatham M, Medenilla A (2023). Performance of ChatGPT on USMLE: Potential for AI-assisted medical education using large language models. PLOS Digit Health.

[12] Gilson A, Safranek CW, Huang T, Socrates V, Chi L, Taylor RA, Chartash D (2023). How does ChatGPT perform on the United States Medical Licensing Examination? The implications of large language models for medical education and knowledge assessment. JMIR Med Educ.

[13] (2023). National Medical Commission: Competency based undergraduate curriculum for the Indian Medical Graduate Volume I. https://www.nmc.org.in/wp-content/uploads/2020/01/UG-Curriculum-Vol-I.pdf.

[14] (2023). Designing multiple-choice questions. https://uwaterloo.ca/centre-for-teaching-excellence/catalogs/tip-sheets/designing-multiple-choice-questions.

[15] Brame C (2023). Brame C: Writing good multiple choice test questions. https://cft.vanderbilt.edu/guides-sub-pages/writing-good-multiple-choice-test-questions/.

[16] Subramani M, Jaleel I, Krishna Mohan S (2023). Evaluating the performance of ChatGPT in medical physiology university examination of phase I MBBS. Adv Physiol Educ.

[17] Friederichs H, Friederichs WJ, März M (2023). ChatGPT in medical school: How successful is AI in progress testing?. Med Educ Online.

[18] Johnson D, Goodman R, Patrinely J (2023). Assessing the accuracy and reliability of AI-generated medical responses: An evaluation of the Chat-GPT model. Res Sq.

[19] Huh S (2023). Are ChatGPT’s knowledge and interpretation ability comparable to those of medical students in Korea for taking a parasitology examination?: A descriptive study. J Educ Eval Health Prof.

[20] van de Ridder JM, Shoja MM, Rajput V (2023). Finding the place of ChatGPT in medical education. Acad Med.

[21] Ali R, Tang OY, Connolly ID (2023). Performance of ChatGPT, GPT-4, and Google Bard on a neurosurgery oral boards preparation question bank [PREPRINT]. Neurosurgery.

[22] Rahsepar AA, Tavakoli N, Kim GH, Hassani C, Abtin F, Bedayat A (2023). How AI responds to common lung cancer questions: ChatGPT vs Google Bard. Radiology.

[23] Kumar D, Jaipurkar R, Shekhar A, Sikri G, Srinivas V (2021). Item analysis of multiple choice questions: A quality assurance test for an assessment tool. Med J Armed Forces India.

